# Stimulatory Effects of Arsenic-Tolerant Soil Fungi on Plant Growth Promotion and Soil Properties

**DOI:** 10.1264/jsme2.ME11316

**Published:** 2012-10-05

**Authors:** Pankaj Kumar Srivastava, Belle Damodara Shenoy, Manjul Gupta, Aradhana Vaish, Shivee Mannan, Nandita Singh, Shri Krishna Tewari, Rudra Deo Tripathi

**Affiliations:** 1 National Botanical Research Institute (CSIR-NBRI), Rana Pratap Marg, Lucknow, 226001 India; 2 Microbial Type Culture Collection and Gene Bank, Institute of Microbial Technology (CSIR-IMTECH), Sector 39A, Chandigarh, 160036 India

**Keywords:** pea, plant growth-promoting fungi, rice, soil enzymes, arsenic-contaminated soils

## Abstract

Fifteen fungi were obtained from arsenic-contaminated agricultural fields in West Bengal, India and examined for their arsenic tolerance and removal ability in our previous study. Of these, the four best arsenic-remediating isolates were tested for plant growth promotion effects on rice and pea in the present study. A greenhouse-based pot experiment was conducted using soil inocula of individual fungi. The results indicated a significant (*P*<0.05) increase in plant growth and improvement of soil properties in inoculated soils compared to the control. A significant increase in plant growth was recorded in treated soils and varied from 16–293%. Soil chemical and enzymatic properties varied from 20–222% and 34–760%, respectively, in inoculated soil. Plants inoculated with inocula of *Westerdykella* and *Trichoderma* showed better stimulatory effects on plant growth and soil nutrient availability than *Rhizopus* and *Lasiodiplodia*. These fungi improved soil nutrient content and enhanced plant growth. These fungi may be used as bioinoculants for plant growth promotion and improved soil properties in arsenic-contaminated agricultural soils.

Chemical fertilizers, agrochemicals and pesticides have been intensively applied to soils to increase crop production to meet food demands all over the world; however, these are disruptive to soil biological processes. Exploring novel microorganisms is important in agriculture to promote soil nutrient cycling and to reduce dependence on inorganic fertilizers. Plant growth-promoting microbes have the ability to convert nutritionally important elements from their unavailable to available form (28) and so their bioavailability increases uptake and furthers plant growth promotion (13). Rhizosphere-competent fungi that exert beneficial effects on plant growth and development are referred to as plant growth-promoting fungi (PGPF). In recent years, the use of PGPF to promote plant growth has increased (4, 18); however, the selection of effective PGPF is critical for maximizing the benefits of using this technology. Several microbes have been isolated from soil, inoculated into rhizospheric soil and tested for their plant growth-promoting ability (24). Better understanding of the interactions between rhizospheric fungi and plants is necessary for the sustainable management of soil fertility *vis-a-vis* increasing crop production (12). Certain fungal species of the genera *Fusarium*, *Penicillium*, *Phoma*, *Trichoderma* as well as other non-sporulating fungi isolated from the plant rhizosphere have been shown to promote the growth of several plants and suppress diseases (10, 30). The mechanisms of plant growth stimulation by associative soil microbes are mainly through the mineralization of nutrients (14), stimulation of root growth by the production of phytohormones (4, 18), *etc*. Phosphorus uptake by plants can be increased by inoculation with soil mineral-phosphate-solubilizing fungi (23). Inoculation with *Penicillium* and *Trichoderma* spp. has increased the growth and yield of wheat and other crop species (36). *Fusarium equiseti* was tested for the control of crown and root rot of tomato in soil during long-term cultivation (10). Medina *et al.* (19) revealed that plant growth, nutrient uptake and soil enzymatic activities were increased in soil inoculated with native mycorrhizal fungi and plant growth-promoting yeast. Similarly, the effect of phosphate-solubilizing fungi (*Aspergillus awamori* and *Penicillium citrinum*), isolated from crop rhizosphere, was observed on the growth and seed production of chickpea plants in pot experiments conducted by Mittal *et al.* (20).

Little is known about the efficiency of metal (loid)-tolerantcum- remediating microbes on plant growth promotion. Recently, some metal (loid)-tolerant microbial strains were isolated from metal (loid)-contaminated soils and evaluated for their role in promoting plant growth (5, 15). In Southeast Asia, elevated arsenic levels in soils are of considerable concern due to the possibility of its uptake by plants and subsequent entry into the food chain. The authors of the study isolated natural fungal populations from arsenic-contaminated agricultural soils in the state of West Bengal, India and tested their arsenic removal efficiency (27). In the present study, the plant growth-promoting effects of the four best arsenic-remediating fungal isolates (27) were evaluated on rice and pea plants. Changes occurring in rhizospheric soil treated with inocula of these fungi were also studied simultaneously in the same greenhouse-based pot experiment.

## Materials and Methods

In our previous study, fifteen fungal strains were isolated from paddy rhizospheres of arsenic-contaminated (range 9.45–15.63 mg kg^−1^) agricultural fields in the state of West Bengal, India (27). Of these fifteen fungal strains, four isolates, frequently found in soil samples in the fields, were found to be tolerant to high levels of arsenic, such as 10,000 mg L^−1^, and were best for arsenic removal under laboratory conditions (27).

### Identification of the fungal isolates

The fungal isolates were grown on potato dextrose agar (PDA) medium for 7 days at 27±2°C and their morphological and microscopic features were recorded using a Trinocular Upright Microscope (Olympus, Tokyo, Japan) (data not shown). Fungal isolates: FNBR-3, FNBR-6 and FNBR-19, were found to produce conidial structures on PDA medium, while fungal isolate FNBR-13 failed to sporulate on PDA medium. This was followed by the isolation of total genomic DNA using the ZR Fungal/Bacterial DNA Kit (Zymo Research, Irvine, CA, USA). DNA quality was checked on 1% (w/v) agarose electrophoresis gel stained with ethidium bromide. The ITS4-ITS5 primer-pair (33, 35) was used to amplify the 5.8S rRNA gene and flanking internal transcribed spacers (ITS) 1 and 2 regions. Amplification reactions were performed in a 50 μl reaction volume as outlined in Shenoy *et al.* (25). The PCR thermal cycle was programmed as follows: 95°C for 3 min, followed by 34 cycles of denaturation at 95°C for 1 min, annealing at 52°C for 30 s and elongation at 72°C for 1 min, with a final extension step of 72°C for 10 min. The PCR products spanning approximately 600 bp were checked on 1% (w/v) agarose electrophoresis gel stained with ethidium bromide. PCR products were then checked using a quick spin column and buffers according to the manufacturer’s protocol (QIA quick gel extraction kit, Qiagen, Hilden, Germany). DNA sequencing was performed using the above mentioned primers in an Applied Biosystem 3130xl analyzer at the Central DNA sequencing facility of the Institute of Microbial Technology (Chandigarh, India). Sequences obtained from the respective primers were aligned using Sequencher version 4.9 (Gene Codes, Ann Arbor, MI, USA). A dataset based on the ITS/5.8S rRNA gene-sequence data was prepared for each fungal strain using reference sequences from NCBI-GenBank. Phylogenetic analyses were conducted based on the maximum likelihood method using MEGA version 5.02 (29).

### Preparation of inoculum

Individual fungal isolates were pure-cultured on rose bengal chloramphenicol agar media (26) and pure mass-cultured in Mycological Broth (Hi-media, Mumbai, India). Individual fungal inoculum was prepared from freshly grown 7-d-old respective pure culture plates by suspending the biomass in sterile distilled water and further mixed by vortex shaking. Inoculum density was adjusted to 10^6^ spores mL^−1^ or mycelial fragments mL^−1^ using a hemocytometer. In the study, soil was collected from the field of the National Botanical Research Institute, Lucknow, India (at 80°45′ to 80°53′E longitude and 26°40′ to 26°45′N latitude, at an elevation of 129 m above mean sea level). This soil was sandy clay loam with pH 7.81 and contained 182 mg g^−1^ total organic carbon, 360 mg g^−1^ total nitrogen, 82 mg g^−1^ total phosphorus and 1.48 cmol kg^−1^ cation exchange capacity.

### Greenhouse Experiment

Two plants, rice (*Oryza sativa* L. *var*. IR-36) and pea (*Pisum sativum* L. *var*. PG-3) were used to assess the plant growth-promoting activity of these fungal isolates in a pot experiment under greenhouse conditions; 500 g soil was used per pot. Pots (size: 20 cm diameter and 25 cm height) were arranged in a completely randomized design with six replicates for each treatment. The seeds were surface sterilized with 1% (w/v) sodium hypochlorite solution followed by thorough washing using sterile distilled water. The roots of rice seedlings and seeds of pea were soaked for 30 min in fungal suspension before sowing in pots. Each pot was planted separately with five one-week-old seedlings in the case of rice and with five seeds in the case of pea and then placed in the greenhouse. After one week, seedlings were thinned to three per pot. Plants were grown under a natural photoperiod under greenhouse conditions with average temperatures of 8°C (night) to 22°C (day), 65–70% relative humidity during the winter season. Plants were harvested after eight weeks. A control set without fungal inocula for both crops was maintained under identical conditions. Manual weeding and watering were performed regularly throughout the experiment. Sterilized water was used for watering the set. No extra fertilizer or manure was added to experimental pots.

### Assessment of plant growth promotion

Plants were harvested eight weeks after planting. Plant root-shoot length and dry biomass were measured. To measure plant biomass, they were dried at 105°C for 24 h. The numbers of tillers/leaves, pods, and stipules produced were recorded. The total chlorophyll, carotenoid (17) and protein contents (16) of the plant leaves were also measured.

### Soil chemical and enzyme assays

Soil samples were taken at the time of plant harvest. Rhizospheric soil samples were kept air-dried and sieved (2 mm) for further analysis in triplicate for each sample. Among various parameters, total organic carbon (TOC) (11) and microbial biomass carbon (MBC) using the chloroform-fumigation-extraction method (32) were assessed. Soil samples had been previously extracted in 2% (w/v) Na-bicarbonate solution to determine the available phosphorus (P) (21). Available potassium (K) was determined using a flame photometer (Systronix-128, Systronix, Salt Lake City, Utah, USA) after extracting samples in 2% (w/v) ammonium acetate solution (11).

Selected soil enzymatic activities were determined in each freshly grounded and sieved (<2 mm) rhizosphere soil sample using four replicates. Dehydrogenase activity (DHA) was assayed following the method of Pepper *et al.* (22) by the reduction of 2,3,5-triphenyl tetrazolium chloride (TTC) and expressed in μg triphenyl formazan (g soil)^−1^h^−1^. Alkaline phosphatase activity (7) and β-glucosidase activity (8) were measured using the substrate analogue paranitrophenyl- β-D-glucopyranoside (pNPG) based on determining the quantity of released p-nitrophenol after incubation of each gram of soil with pNPG solution for 1 h at 37°C.

### Re-isolation of fungal isolates

Re-isolation of inoculated fungi was carried out to check the plant pathogenicity of tested isolates. For epiphytic isolation, the root segments were washed using sterile water, cut into 1 cm long segments aseptically, and then incubated on PDA supplemented with a sterile commercial solution (100×) containing penicillin and streptomycin in 0.9% NaCl (Hi-media, Mumbai, India). The colonies emerging from root segments were identified morphologically and microscopically by comparing their characteristics with those of tested original fungal colonies on PDA (data not shown). In addition, re-isolation was carried out under sterilized conditions to check the endophytic nature of the fungal isolates. For endophytic isolation, the harvested roots were cut into 5 cm long segments, washed in sterile water and then disinfected with 0.1% HgCl_2_ solution for 30 s followed by five washings with sterilized water to remove traces of HgCl_2_. The segments were air dried under aseptic conditions, after which some segments were placed on PDA, pressed gently and removed after 30 s. Re-occurrence of the tested isolates was checked to confirm the pathogenic nature of tested fungi.

### Statistical analysis

The experimental data were subjected to analysis of variance (ANOVA) with the Duncan Multiple Range Test (DMRT) using significance at *P*<0.05 with the SPSS statistical package for Windows version 10.0 (SPSS, Chicago, IL, USA).

### Nucleotide sequence accession numbers

The ITS/5.8S rRNA gene sequence data have been deposited in NCBI-GenBank with accession numbers JN102303 and JN118571– JN118573. The accession URL for their phylogenies is http://purl.org/phylo/treebase/phylows/study/TB2:S12359.

## Results and Discussion

The results of the present study indicate the plant growth-promoting ability of tested fungal isolates under greenhouse conditions. Based on morphology, microscopic features (data not shown) and phylogenetic analysis of the ITS region, these fungal isolates have been identified as *Westerdykella aurantiaca* FNBR-3, *Trichoderma longibrachiatum* FNBR- 6, *Lasiodiplodia* sp. FNBR-13 and *Rhizopus delemar* FNBR-19. Taxonomically, *Westerdykella aurantiaca* FNBR-3 belongs to family—*Sporormiaceae*, order—*Pleosporales* of *Ascomycota*; *Trichoderma longibrachiatum* FNBR-6 to family— *Hypocreaceae*, order—*Hypocreales* of *Ascomycota*; *Lasiodiplodia* sp. FNBR-13 to family *Botryosphaeriaceae*, order—*Botryosphaeriales* of *Ascomycota*; and *Rhizopus delemar* FNBR-19 to family—*Mucoraceae*, order—*Mucorales* of *Zygomycota*. Maximum likelihood trees generated in this study were registered in “Treebase” and the fungal isolates have been deposited in the Microbial Type Culture Collection and Gene Bank, Chandigarh, India (http://mtcc.imtech.res.in) with accession numbers MTCC 10845- 10848. The re-isolated fungal colonies emerging from sterilized root segments were morphologically and microscopically different from the inoculated segments (data not shown), ruling out the possibility of the tested isolates being endophytic and pathogenic in nature. Identification of the reisolates other than the tested inoculants was not attempted.

**Fig. 1:**
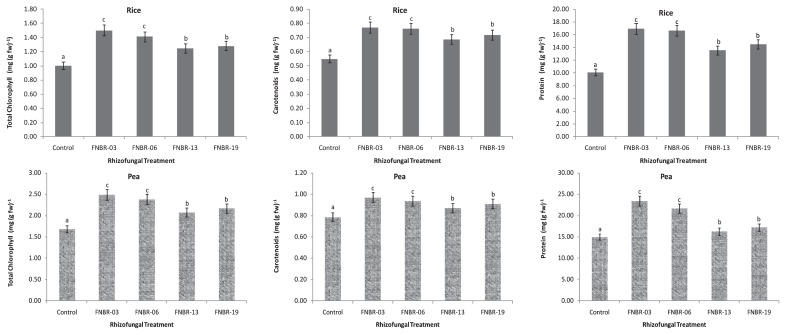
Effects of rhizofungal inoculum on total chlorophyll, carotenoid and protein contents (mg g^−1^ fresh weight) of plant leaves. Values (mean ± SD) of a line followed by the same letter are not significantly different according to the Duncan Multiple Range Test (*P*<0.05).

Changes in chemical and biochemical properties of plant rhizospheric soil most probably provoked by the fungal inoculum are shown in Table 1. These strains stimulated TOC and MBC contents of soil significantly (*P*<0.05) with an increase in the range of 34–222% and 53–460%, respectively. The inoculated fungi also increased the available K content of soil in the significant range of 20–119% and available P content as 18–110% compared to the control. Soil enzyme activities were also higher in the rhizosphere of inoculated plants than in un-inoculated controls. Inoculation of these fungal strains had a significant stimulating effect on the soil enzyme activities. The increase in DHA was 34–154% and 132–422% for alkaline phosphatase, and 262–760% for β-glucosidase activity in all four inoculated fungi. Fungal strains FNBR-3 and FNBR-6 performed better than the other two strains (FNBR-13 and FNBR-19) in provoking soil chemical properties as well as the enzymatic activities of inoculated soil. The results are presented in Table 2, showing the plant growth-promoting effects of the fungal inoculum on the growth of rice and pea plants grown for eight weeks. Inoculation of plant rhizospheres with these fungal strains significantly (*P*<0.05) increased root length (25–85%), shoot length (20–82%), number of tillers (31–100%), and plant dry weight (47–250%) in the case of rice. A significant increase in root length (20–81%), shoot length (16–100%), number of stipules (22–84%), number of leaves (28–113%), number of pods (83–293%), and plant dry biomass (50–152%) was also observed in pea. An increase in total plant dry matter of tested plants (in the case of rice as 104 and 250%; and in pea as 103 and 152% with FNBR-3 and FNBR-6, respectively) showed the efficiency of these two isolates over FNBR-13 and FNBR-19. FNBR-13 and FNBR-19 strains also stimulated plant growth, but without any comparable significant difference between their effects. Quantitative assessment of the biochemical properties of plant leaves also showed that all the fungal isolates inoculated plants significantly (*P*<0.05), increasing the total chlorophyll, carotenoids and protein contents (Fig. 1). Strains FNBR-13 and FNBR- 19 increased these biochemical parameters of plant leaves without any comparable significant difference between their effects. Fungal strains FNBR-3 and FNBR-6 produced almost identical effects. Presumably, the plant growth increase and soil improvement could be observed due to the activities of PGPF through the production and release of secondary metabolites (plant growth regulators, phytohormones and biologically active substances), preventing the effects of phyto-pathogenic organisms in the rhizosphere and/or facilitating the availability and uptake of soil nutrients from the root environment (4, 18, 30). These mycelial exudates seem to play an important role in plant growth promotion. According to Viterbo *et al.* (34), *Trichoderma* can produce 1-aminocyclopropane-1-carboxylate (ACC) deaminase, which can contribute to plant growth promotion, such as an increase in root elongation. These isolates did not have a negative impact on plants or soil, and no disease symptoms appeared on the tested plants.

Soil fungi have been shown to exhibit plant growth-promoting activity on numerous cultivated plants (4, 18). Previously, Saldajeno *et al.* (23) discussed the significant plant growth-promoting effects and yield induced by inoculated PGPF. The maximum stimulatory effect on chickpea plant growth was observed by inoculation of *Aspergillus* and *Penicillium* strains and resulted in an incremental increase in shoot height, a nearly three-fold increase in seed number and two-fold increase in seed weight as compared to the control (un-inoculated) plants (20). Shoot and root plant biomass, values of thymidine and leucine incorporation as well as ergosterol and chitin in rhizosphere soil increased due to fungal metabolic activity and fungal biomass (19). Such microbial inoculation could compensate for nutrient deficiency and improve plant development through the production of plant growth regulators by microbes at the root interface (30). This phenomenon could stimulate the root development of plants and result in better absorption of water and nutrients from the soil. The beneficial effects on plant growth observed as a result of the inoculation of PGPF may not be related only to the stimulation of colonization by these fungal isolates in plant rhizospheres, but an increase in the development of the extra-radical mycelium could also be involved. Improved soil structure might be achieved through the secretion of mucilage/polysaccharides by inoculated fungi, which could further improve soil aggregation (2). This might be the reason for the higher concentrations of TOC in the study. Effectiveness of these treatments on plant growth could also be due to the indirect effect through changes in microbial composition in the rhizosphere (19). Qualitative and quantitative changes in soil rhizospheric microbial populations due to bioinoculants have also been estimated through the measurement of soil enzyme activity. The increased enzyme activities found in the rhizosphere of fungus-inoculated plants might be an indication of the increase in carbon and nutrient mineralization in the study. The measurement of hydrolases provides an indication of changes in soil fertility, since they are related to the mineralization of important nutrient elements required for both plant and microbial growth (1). Application of biofertilizer increased β-glucosidase, phosphatase activities, soil aggregate stability, water-soluble carbohydrates and plant phosphorus acquisition in inoculated soils (19). Many soil fungi have been shown to possess the ability to solubilize sparingly soluble phosphates *in vitro* by secreting inorganic or organic acids (36). George *et al.* (9) also indicated that the fungal phytases increased hydrolysis of inositol phosphates in soil environments. Likewise, Kim *et al.* (14) suggested that the secretion of phosphatases by phosphate-solubilizing fungi frequently facilitates the conversion of insoluble forms of phosphate to plant-available forms and enhances plant phosphorus uptake and further growth of the treated plants. Microbe-inoculated plants showed an increased root system that, in turn, increased carbon and nutrient inputs to the rhizosphere zone. In the study, activities of glucosidase and phosphatase improved significantly in the rhizosphere of inoculated plants with more growth. Caravaca *et al.* (3) also studied that the soil had higher levels of available phosphorus, aggregate stability, total carbohydrates, biomass carbon and water-soluble carbon and enzyme activities (dehydrogenase, phosphatase and β-glucosidase) in the rhizosphere of PGPF-inoculated plants than in control plants. In the same study, shoot biomass of PGPF-inoculated *Dorycnium pentaphyllum* L. plants was about 219% greater with respect to control plants. The solubilization of soil nutrients and their increased uptake have also been demonstrated by de Silva *et al.* (6) for soil-inhabiting fungal biocontrol agents such as *Gliocladium virens* and *Trichoderma harzianum*. Turpeinen *et al.* (31) suggested that metal-tolerant microbes can maintain their metabolic activities in contaminated soils. These fungi are able to tolerate, biosorb and detoxify arsenic by several mechanisms, including valence transformation, extra- and intracellular precipitation, active uptake and methylation (27). These results encourage mesocosmic research experiments that may help to study the growth promotion of crops inoculated with these arsenic-tolerant fungal strains in arsenic-contaminated soils. Using these fungal strains could be a realistic and desirable strategy for maintaining crop production in arsenic-contaminated soils as well as mitigating arsenic contamination of crops. Nevertheless, it is evident that additional research will be necessary to identify the application range of these arsenic-tolerant and plant growth-promoting fungal strains in terms of soil types, competitiveness and ecology for their ‘in-situ application’.

## Conclusion

The use of plant growth-promoting rhizofungus (PGPF) as a bioinoculant is a recent area of interest. Metal (loid)- tolerant and metal (loid)-remediating PGPF inoculants can play an important role in maintaining sustainable agricultural production in metal (loid)-contaminated soils. The results of the study reveal the plant growth-promoting efficiency of four arsenic-tolerant and -remediating fungi along with their positive effects on soil nutritive properties. The stimulatory efficiency of tested fungal strains on plant growth and soil properties showed their potential to be utilized as bio-culture for better crop cultivation in arsenic-contaminated agricultural soils.

## Figures and Tables

**Table 1:** Effects of rhizofungal inoculum on chemical and biochemical properties of rhizospheric soil of rice and pea plants grown for eight weeks

Soil Properties	Rice					Pea				
	Control	FNBR-3	FNBR-6	FNBR-13	FNBR-19	Control	FNBR-3	FNBR-6	FNBR-13	FNBR-19
Dehydrogenase activity [μg TPF (g soil)^−1^ h^−1^]	0.993^a^±0.013	2.36^c^±0.41	2.56^c^±0.72	1.54^b^±0.73	1.92^b^±0.78	1.009^a^±0.033	2.451^c^±0.265	2.563^c^±0.011	1.923^b^±1.169	2.014^b^±0.146
Alkaline phosphatase activity [μg þ-NP (g soil)^−1^ h^−1^]	27.54^a^±1.36	119.85^c^±17.28	140.97^d^±1.75	63.91^b^±6.04	72.00^b^±10.82	28.22^a^±0.43	124.49^c^±14.69	134.71^d^±6.48	66.35^b^±2.06	71.44^b^±10.01
β-glucosidase activity [μg þ-NP (g soil)^−1^ h^−1^]	2.13^a^±0.94	14.51^c^±2.48	18.31^d^±1.69	9.64^b^±1.94	7.95^b^±0.56	2.78^a^±1.77	12.17^c^±2.39	18.40^d^±0.25	10.07^b^±0.26	8.43^b^±1.76
Total organic carbon (TOC) (%)	0.180^a^±0.023	0.306^c^±0.06	0.551^d^±0.06	0.241^b^±0.001	0.281^b^±0.095	0.180^a^±0.023	0.316^c^±0.18	0.580^d^±0.114	0.280^b^±0.023	0.342^b^±0.115
Microbial biomass carbon (MBC) [μg (g soil)^−1^]	65.89^a^±3.39	218.3^c^±20.34	280.58^d^±18.81	100.86^b^±12.68	121.05^b^±19.14	77.47^a^±3.82	347.97^c^±36.32	433.62^d^±33.42	139.18^b^±56.85	218.04^b^±27.08
Available phosphorus [g (kg soil)^−1^]	80.02^a^±8.78	130.12^c^±4.68	168.13^d^±14.78	95.11^b^±1.65	102.72^b^±20.42	84.53^a^±3.27	142.44^c^±2.46	179.03^d^±10.55	100.98^b^±15.93	115.55^b^±8.05
Available potassium [g (kg soil)^−1^]	23.07^a^±4.71	33.75^c^±8.38	37.37^d^±4.09	27.68^b^±4.32	30.65^b^±1.77	24.45^a^±3.41	41.30^c^±8.61	53.65^d^±5.91	38.62^b^±6.28	37.4^b^±4.14

Values (mean ± SD) within a row for each plant followed by the same letter are not significantly different according to the Duncan Multiple Range Test (*P*<0.05).

**Table 2:** Effects of rhizofungal inoculum on the growth of rice and pea plants grown for eight weeks

Plants	Plant Growth Parameters	Control	FNBR-3	FNBR-6	FNBR-13	FNBR-19
Rice	Root length (cm)	15.28^a^±3.67	25.01^c^±4.03	28.27^c^±1.97	19.11^b^±1.51	21.66^b^±1.16
	Shoot length (cm)	25.40^a^±1.45	40.85^c^±2.82	46.28^d^±1.19	30.38^b^±1.25	34.56^b^±2.14
	Number of tillers	8.25^a^±1.95	14.75^c^±2.06	16.75^d^±2.62	10.80^b^±1.63	11.50^b^±2.38
	Plant dry biomass (g plant^−1^)	1.077^a^±0.564	2.206^c^±1.054	3.778^d^±0.887	1.587^b^±0.983	1.942^b^±0.764
Pea	Root length (cm)	10.75^a^±1.77	18.58^c^±3.22	19.45^c^±2.27	12.90^b^±1.79	14.55^b^±2.62
	Shoot length (cm)	20.49^a^±2.15	33.93^c^±4.48	41.02^d^±4.92	23.75^b^±2.62	25.54^b^±3.61
	Number of stipules	8.00^a^±2.35	12.75^c^±3.19	14.75^d^±3.66	9.80^b^±3.96	10.50^b^±2.51
	Number of leaves	13.75^a^±2.36	25.25^c^±1.78	29.25^d^±1.03	17.55^b^±1.68	20.50^b^±1.21
	Number of pods	1.50^a^±0.95	4.75^c^±1.41	5.90^d^±1.15	2.75^b^±0.88	2.75^b^±0.95
	Plant dry biomass (g plant^−1^)	3.85^a^±1.05	7.85^c^±2.95	9.71^d^±2.04	5.78^b^±1.54	6.41^b^±2.77

Values (mean ± SD) within a row followed by the same letter are not significantly different according to the Duncan Multiple Range Test (*P*<0.05)
